# A suppressive role of ionizing radiation-responsive miR-29c in the development of liver carcinoma via targeting *WIP1*

**DOI:** 10.18632/oncotarget.3157

**Published:** 2015-04-04

**Authors:** Bo Wang, Dongping Li, Corinne Sidler, Rocio Rodriguez-Juarez, Natasha Singh, Mieke Heyns, Yaroslav Ilnytskyy, Roderick T. Bronson, Olga Kovalchuk

**Affiliations:** ^1^ Department of Biological Sciences, University of Lethbridge, Lethbridge, Canada; ^2^ Department of Biochemistry, Qiqihar Medical University, Qiqihar, P.R. China; ^3^ The Dana Farber/Harvard Comprehensive Cancer Center, Boston, Massachusetts, USA

**Keywords:** ionizing radiation, miR-29c, hepatocellular carcinoma, WIP1

## Abstract

Hepatocellular carcinoma (HCC) is the third most common cause of cancer-related deaths worldwide, and it has been linked to radiation exposure. As a well-defined oncogene, wild-type p53-induced phosphatase 1 (WIP1) plays an inhibitory role in several tumor suppressor pathways, including p53. *WIP1* is amplified and overexpressed in many malignancies, including HCC. However, the underlying mechanisms remain largely unknown. Here, we show that low-dose ionizing radiation (IR) induces miR-29c expression in female mouse liver, while inhibiting its expression in HepG2, a human hepatocellular carcinoma cell line which is used as a model system in this study. miR-29c expression is downregulated in human hepatocellular carcinoma cells, which is inversely correlated with WIP1 expression. miR-29c attenuates luciferase activity of a reporter harboring the 3′UTR binding motif of *WIP1* mRNA. Ectopic expression of miR-29c significantly represses cell proliferation and induces apoptosis and G1 arrest in HepG2. In contrast, the knockdown of miR-29c greatly enhances HepG2 cell proliferation and suppresses apoptosis. The biological effects of miR-29c may be mediated by its target WIP1 which regulates p53 activity via dephosphorylation at Ser-15. Finally, fluorescence *in situ* hybridization (FISH) and immunohistochemical analyses indicate that miR-29c is downregulated in 50.6% of liver carcinoma tissues examined, whereas WIP1 is upregulated in 45.4% of these tissues. The expression of miR-29c inversely correlates with that of WIP1 in HCC. Our results suggest that the IR-responsive miR-29c may function as a tumor suppressor that plays a crucial role in the development of liver carcinoma via targeting *WIP1*, therefore possibly representing a target molecule for therapeutic intervention for this disease.

## INTRODUCTION

HCC is the most common primary liver malignancy in adults and the third most common cause of cancer-related deaths worldwide [1, 2]. In Canada, HCC is a growing problem. Based on data from the Canadian Cancer Registry, age-adjusted liver cancer incidence rose to 15.4 per 100,000 for the period 2006–2010 [3], three times the incidence for the period 1976–1980. The primary risk factors for HCC may be chronic hepatitis B (HBV) and C virus (HCV) infections and cirrhosis because 78% of HCC cases and 57% of liver cirrhosis cases are caused by chronic infection with HBV and HCV [4]. Most solid cancers have been linked to radiation [5], including liver cancer. The dose-response relationship between radiation and liver cancer has been clinically verified in primary liver cancer cases and implicated in histological analyses [6].

MicroRNAs (miRNAs/miRs) are small noncoding RNA molecules that negatively regulate gene expression at the post-transcriptional level through either translational repression or mRNA cleavage [7, 8]. Approximately 60% of human protein-coding genes are targets of miRNAs [9]. These molecules may function as tumor suppressors or oncogenes [10–12] that play crucial roles in diverse biologic and pathologic processes, such as cell proliferation, differentiation, and apoptosis [7, 13]. miRNAs are also involved in genotoxic stress responses. We have recently shown that IR causes a profound dysregulation of miRNA expression in exposed tissues and organs, such as the thymus, mammary gland, and liver. Among miRNAs, miR-29c is significantly influenced in the IR-exposed liver tissue.

miR-29c belongs to the miR-29 family which includes three other members in humans: miR-29a, miR-29b-1, and miR-29b-2 [14]. Since miR-29b-1 and miR-29b-2 have identical mature sequences, they are collectively called miR-29b [14]. miR-29a and miR-29b-1 are transcribed together as a polycistronic primary transcript [15, 16], while miR-29b-2 and miR-29c are transcribed together. The members of this family may function as tumor suppressors, and a loss of function of these miRNAs may contribute to tumorigenesis and metastasis. A large body of evidence has demonstrated that miR-29c is frequently reduced in human malignancies, including gastric cancer [17], metastatic lung cancer [18], chronic lymphocytic leukemia [19], metastatic medullary thyroid carcinoma [20], peripheral nerve sheath tumors [21], hepatocellular carcinoma [22, 23], meningioma [24], and nasopharyngeal [25, 26] and esophageal squamous cell carcinomas [27].

WIP1 (also known as PP2Cδ), a nuclear serine/threonine phosphatase encoded by *protein phosphatase magnesium-dependent 1 delta* (*PPM1D*) [28] belongs to the Ser/Thr PP2C family of phosphatases. The members in this evolutionarily conserved family are key players in the regulation of cellular stress response [28, 29]. WIP1 is not only a direct transcriptional target of tumor suppressor p53 but also an important negative regulator of p53, thus forming a negative regulatory feedback loop [30, 31]. The negative regulatory effect of WIP1 on p53 may be primarily attributed to its ability to dephosphorylate p53 Ser-15 [32].

miR-29c was bioinformatically predicted to target WIP1. However, the expression of miR-29c in response to IR in the liver and the role of miR-29c in hepatocellular carcinogenesis are not completely understood yet. We therefore explored the expression of miR-29c in liver tissue of mice exposed to IR and determined the contribution of miR-29c to the development of hepatocellular carcinoma. The data presented in this paper indicate that low-dose IR triggers a profound induction of miR-29c expression in mouse liver tissue. We also show that miR-29c is downregulated in both mouse hepatoma and human hepatocellular carcinoma cells. The ectopic expression of miR-29c inhibits hepatocellular carcinoma cell proliferation and induces apoptosis and G1 cell cycle arrest. In contrast, the knockdown of miR-29c promotes cell proliferation and represses apoptosis. We provide evidence that miR-29c directly targets *wild-type p53-induced phosphatase 1* (*WIP1*). Furthermore, the FISH analysis shows that miR-29c was downregulated in 50.6% of the human hepatocellular carcinoma specimens examined (*n* = 255). Immunohistochemical staining indicates that WIP1 was overexpressed in 45.4% of the hepatocellular carcinoma tissues analyzed (*n* = 249).

## RESULTS

### Low-dose IR triggers a differential expression of miR-29c in female mouse liver and human HepG2 cells

**Figure 1:**
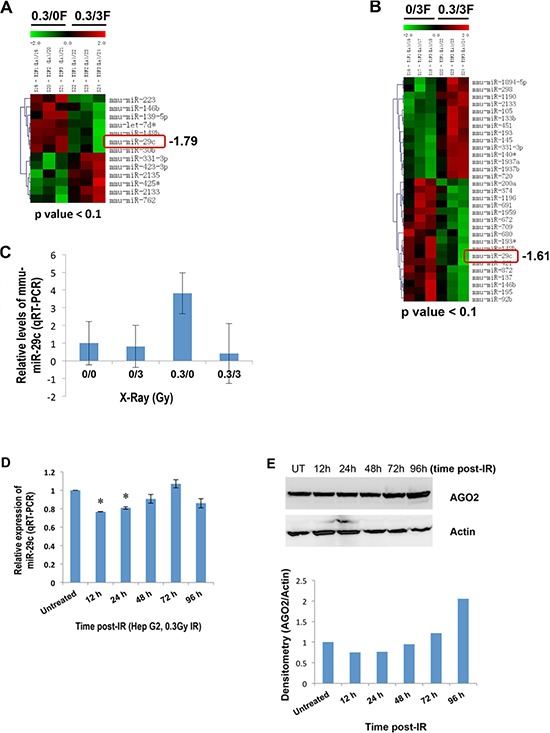
A differential expression of miR-29c in liver tissues of female mice exposed to IR and human HepG2 cells **(A** and **B)** Total RNA isolated from IR-exposed female mouse liver tissues 96 hours post-irradiation was subjected to the microRNA microarray. **(C)** Total RNA was isolated from liver tissues of female mice exposed to IR 96 hours post irradiation, and the levels of miR-29c were examined by real-time RT-PCR. **(D)** Total RNA isolated from human hepatocellular carcinoma HepG2 cells at the indicated time points after exposure to low-dose X-ray (0.3 Gy) was subjected to real-time RT-PCR using miR-29c primer. **(E)** Whole cellular lysate prepared from HepG2 cells at the indicated time points after exposure to low-dose X-ray (0.3 Gy) was subjected to Western blotting with a specific antibody to AGO2. The asterisk indicates *p* < 0.05.

Our previous studies indicated that IR triggered a profound, sex-specific deregulation of microRNAome in the spleen of C57BL/6 mice [33]. To explore miRNAs that are differentially expressed in liver tissues in response to IR, 8-week-old female C57BL/6 mice were exposed to different doses of X-ray and sacrificed 96 hours after irradiation. The microRNA microarray analysis showed that miR-29c was remarkably elevated in response to low-dose IR (Figure 1A and 1B).

These results were validated by quantitative real-time RT-PCR (qRT-PCR, Figure 1C). qRT-PCR also showed a differential expression of miR-29c in the liver tissue of mice exposed to IR at the indicated time points (supplementary Figure S1). To explore an expression pattern of miR-29c in human hepatocellular carcinoma HepG2 cells in response to low-dose IR, HepG2 cells were exposed to 0.3 Gy X-ray, and the expression of miR-29c was then determined. qRT-PCR indicated that low-dose IR had no effect on the expression of miR-29c in HepG2 cells at 96 hours post IR, whereas IR did suppress its expression at 12 and 24 hours after IR (Figure 1D). To understand the mechanism involved, we examined the expression of argonaute RISC catalytic component 2 (AGO2). Western blot analysis showed that AGO2 was downregulated at 12 and 24 hours post IR and upregulated at 96 hours after it (Figure 1E), which may contribute to the IR-responsive miR-29c expression in HepG2 cells.

### *WIP1* is a novel direct target of miR-29c

**Figure 2:**
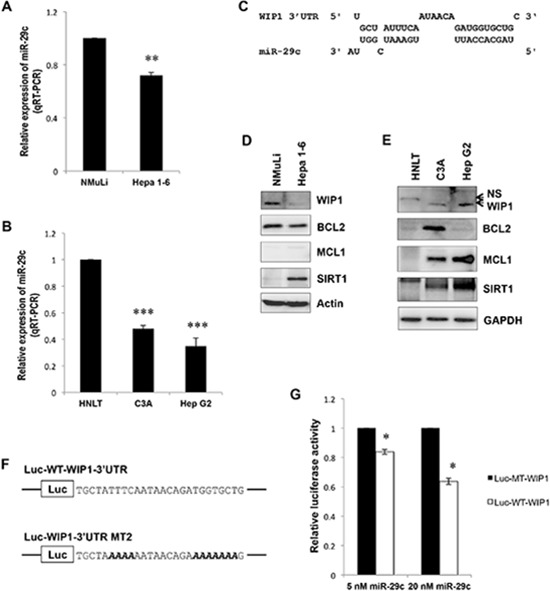
miR-29c is downregulated in liver carcinoma cells and directly targets *WIP1* **(A** and **B)** Total RNA isolated from an epithelial cell line NMuLi derived from normal mouse liver, a mouse hepatoma cell line Hepa 1–6, human hepatocellular carcinoma cell lines HepG2 and C3A, and human normal liver tissue (HNLT) was subjected to real-time RT-PCR analysis using the miR-29c primer assays. **(C)** WIP1, a predicted target of miR-29c. **(D** and **E)** The whole lysate was prepared from NMuLi, Hepa 1–6, C3A and HepG2 cell lines, and HNLT; Western blot analysis was performed using antibodies against BCL2, MCL1, SIRT1, and WIP1. **(F)** Sequences of the wild-type and mutant WIP1 3′UTR reporters were used in this study; bold Italic letters indicate the mutated nucleotides. **(G)** HEK293 cells were transiently transfected with either the Luc-WT-WIP1 or Luc-MT-WIP1 reporter in combination with the indicated concentration of miR-29c; the luciferase activity was detected according to the manufacturer's instructions. The asterisk indicates *p* < 0.05; double asterisks indicate *p* < 0.01; triple asterisks indicate *p* < 0.001.

To determine the role of IR-responsive miR-29c in liver cancer, we measured the expression of miR-29c in hepatocellular carcinoma cells. qRT-PCR showed that miR-29c was significantly downregulated in both mouse (Hepa 1–6) and human (HepG2, C3A) hepatocellular carcinoma cells (Figure 2A and 2B; *p* < 0.01), which was consistent with the previous report [23]. To better understand the role of miR-29c and identify its novel targets, we performed a bioinformatics analysis where *WIP1* was predicted as a potential target of miR-29c (Figure 2C). Western blot analysis showed that WIP1 was upregulated in two of the examined human hepatocellular carcinoma cell lines (Figure 2E), which was inversely correlated with miR-29c expression (Figure 2B), although WIP was downregulated in mouse Hepa 1–6 cells (Figure 2D). To confirm that miR-29c directly targets *WIP1*, we generated luciferase reporters bearing either the wild-type or mutant 3′ UTR of *WIP1* (Figure 2F). The luciferase assay indicated that miR-29c significantly reduced the activity of wild-type WIP1 luciferase in a dose-dependent manner, while this reduction was abolished in the mutant WIP1 reporter (Figure 2G; *p* < 0.05). These results suggest that *WIP1* is a direct target of miR-29c. Because oncogene *sirtuin 1* (*SIRT1*) and two antiapoptotic molecules, *B-cell CLL/lymphoma 2* (*BCL2*) and *myeloid cell leukemia sequence 1* (*MCL1*), have been identified as direct targets of miR-29c [22, 23], we also analyzed their expression in Hepa 1–6, C3A, and HepG2 cell lines. Western blot analysis showed that MCL1 was overexpressed in two human hepatocellular carcinoma cell lines, while SIRT1 was elevated in all the examined cell lines (Figure 2D and 2E).

### miR-29c may function as a tumor suppressor in hepatocellular carcinoma

**Figure 3:**
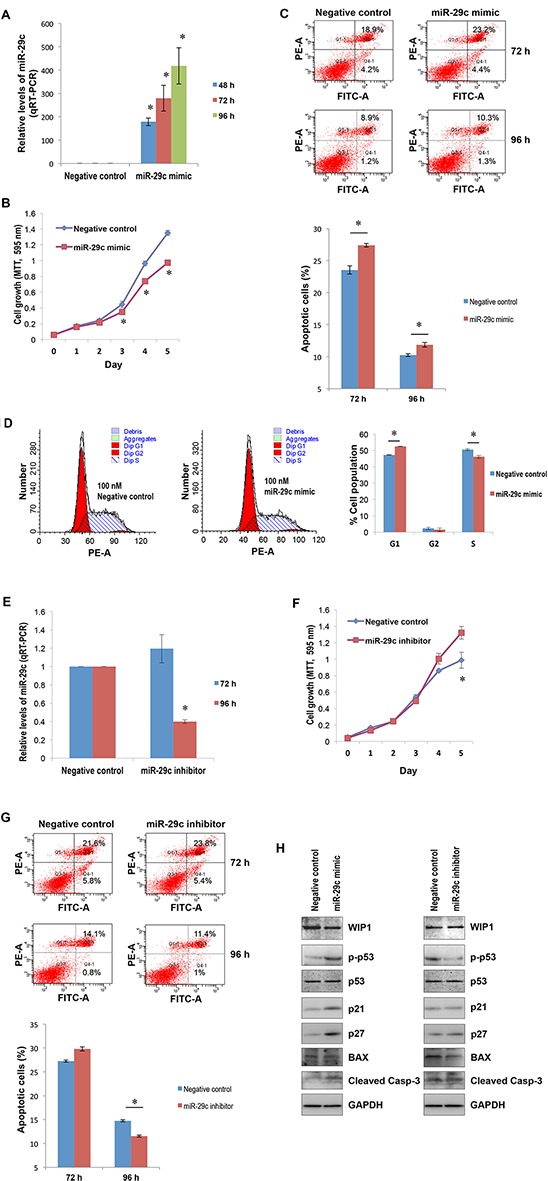
The suppressive role of miR-29c in hepatocellular carcinogenesis **(A)** HepG2 cells were transfected with either 100 nM miR-29c or negative control; total RNA isolated from these cells at the indicated time points was subjected to real-time RT-PCR using the miR-29c primer assays. **(B)** HepG2 cells transfected with either 100 nM miR-29c or negative control were re-plated in 96-well plates; the MTT assay was carried out as described in “Materials and Methods”. **(C)** HepG2 cells transfected with either 100 nM miR-29c or negative control were harvested for apoptosis analysis at the indicated time points as described in “Materials and Methods”; the lower panel represented the average values of independent experiments. **(D)** 96 hours post transfection, HepG2 cells transfected with either 100 nM miR-29c or negative control were harvested for cell cycle analysis as described in “Materials and Methods”. **(E)** HepG2 cells were transfected with either 100 nM miR-29c inhibitor or negative control; total RNA isolated from these cells at the indicated time points was subjected to real-time RT-PCR using the miR-29c primer assays. **(F)** HepG2 cells transfected with either 100 nM miR-29c inhibitor or negative control were re-plated in 96-well plates; the MTT assay was carried out as described in “Materials and Methods”. **(G)** HepG2 cells transfected with either 100 nM miR-29c inhibitor or negative control were harvested for apoptosis analysis at the indicated time points as described in “Materials and Methods”; the lower panel represented the average values of independent experiments. **(H)** 96 hours post transfection, the whole cellular lysate prepared from HepG2 cells transfected with either 100 nM miR-29c mimic (the left panel), miR-29c inhibitor (the right panel), or negative control was subjected to Western blotting with antibodies to BAX, cleaved caspase 3, p21, p27, p53, p-p53 and WIP1. The asterisk indicates *p* < 0.05.

Next, we determined the role of miR-29c in liver carcinogenesis using HepG2 as a model system. With transient transfection, the MTT assay showed that miR-29c significantly suppressed hepatocellular carcinoma cell proliferation (Figure 3A and 3B; *p* < 0.05). The ectopic expression of miR-29c also induced apoptosis and G1 cell cycle arrest (Figure 3C and 3D). Conversely, miR-29c inhibitor significantly promoted liver carcinoma cell proliferation (Figure 3E and 3F; *p* < 0.05) and slightly inhibited apoptosis (Figure 3G), although it had no effect on cell cycle (data not shown). To explore the underlying mechanism, we determined the expression of WIP1 and its target, phosphorylated p53 at Ser-15 [32]. Western blot analysis indicated that miR-29c reduced WIP1 expression (Figure 3H, the left panel), leading to an elevation in phosphorylated p53 at Ser-15. Conversely, miR-29c inhibitor promoted WIP1 expression (Figure 3H, the right panel), resulting in a decrease in the phosphorylated p53. The enforced expression of miR-29c also led to an induction in p21 and p27 expression and an increase in the cleaved caspase 3 (Figure 3H, the left panel). miR-29c inhibitor, however, had no effect on the expression of p21 and p27 and the levels of cleaved caspase 3, but it reduced the expression of BAX (Figure 3H, the right panel). These results suggest that miR-29c plays a suppressive role in hepatocellular carcinoma by targeting WIP1.

### Downregulated miR-29c may contribute to the upregulation of WIP1 in liver carcinoma tissues

**Figure 4:**
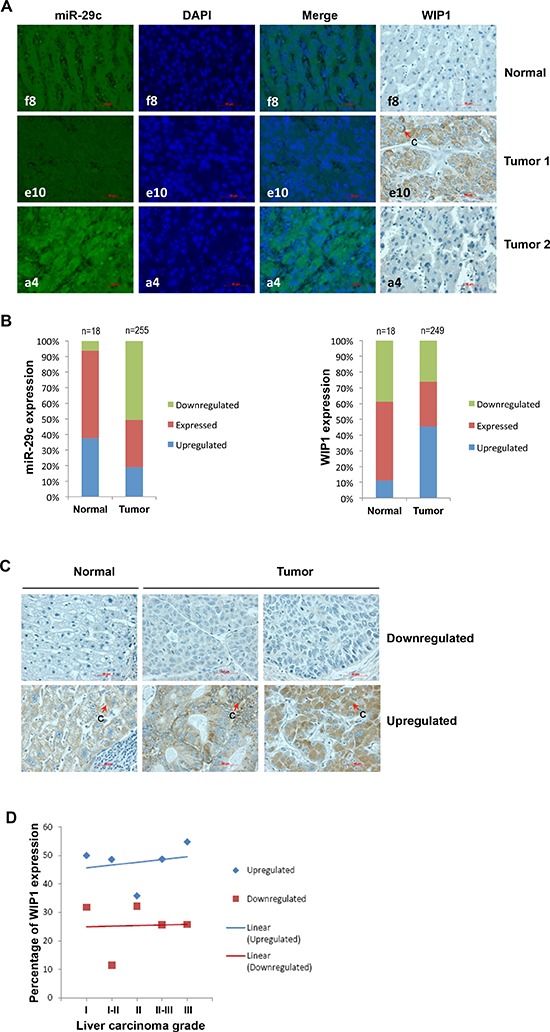
The expression of miR-29c and WIP1 in hepatocellular carcinoma tissues **(A)** Representatives of hsa-miR-29c and WIP1 stainings in the same sections of liver carcinoma tissue arrays. **(B)** Statistical analyses of hsa-miR-29c and WIP1 expression in liver carcinoma tissues. **(C)** Representatives of WIP1 staining in normal liver and carcinoma tissues. **(D)** The frequency of WIP1 expression in the progression of liver carcinoma “c” indicates cytoplasmic staining.

Next we performed the bioinformatics analysis of the correlation between miR-29c and WIP1 levels using a number of publically available deep sequencing datasets. A very strong inverse correlation was found in HeLa cells, although only a weak inverse correlation was observed in three Cancer Genome Atlas datasets (kidney renal clear cell carcinoma, lung squamous cell carcinoma, and rectum adenocarcinoma). To validate the inverse correlation between miR-29c and WIP1 in a large number of samples, we determined the expression of these two molecules in liver carcinoma tissue arrays and subsequently performed a correlation analysis. The FISH analysis showed that miR-29c was downregulated in 50.6% (*n* = 255) of the liver carcinoma tissues examined (Figure 4A and 4B). However, the immunohistochemical analysis revealed that WIP1 was upregulated in 45.4% (*n* = 249) of the liver carcinoma tissues (Figure 4A and 4B). The expression of miR-29c was inversely correlated with that of WIP1 in the tissues (Pearson *r* = −0.8488). The upregulated WIP1 was located primarily in the cytoplasm (Figure 4C). Frequently, WIP1 was overexpressed in the progression of this disease (Figure 4D).

## DISCUSSION

Although low-dose IR has been shown to improve the efficacy of chemotherapeutic agents [34, 35], the potential contribution of low-dose IR to enhancing radiation-induced tumor-cell killing is a matter of debate. It has been indicated that in radiotherapy treatment, smaller fractions (< 0.5 Gy) delivered before larger fractions (> 0.5 Gy) lead to a small increase in cell kill *in vitro* [36]. However, in some *in vivo* studies that used glioblastoma xenografts, three small fractions (0.4 Gy per day) failed to show an improved outcome [37]. Furthermore, low-dose radiation exposure has been linked to an increased cancer risk [38].

This study for the first time reveals that low-dose IR induces miR-29c expression in female mouse liver. Interestingly, we noted that the expression pattern of miR-29c in the IR-exposed human hepatocellular carcinoma cell line HepG2 appeared to differ from that in female liver of mice exposed to IR because it was upregulated in normal female mouse liver and downregulated in liver carcinoma HepG2 cells in response to low-dose IR (Figure 1C and 1D). Our results implicate that low-dose IR may have a protective effect on malignant transformation in normal liver and a promotive effect in liver carcinoma, which argues for the potential of low-dose IR to slightly increase cell killing (including cancer cells) *in vitro* [36]. Although the mechanism is still unclear, alterations in AGO2 expression may contribute to the low-dose IR-triggered differential expression of miR-29c.

We have found that miR-29c is downregulated in liver carcinoma cell lines and tissues, which is consistent with previous reports showing frequent reductions of miR-29c in human malignancies, including hepatocellular carcinomas [22, 23]. It has been shown that the re-induction of miR-29c suppresses cell proliferation [18, 22, 27], migration and invasion [18, 21, 25], induces apoptosis [23] and attenuates tumor xenograft growth *in vivo* [17, 23, 27]. We also provide strong evidence for the tumor-suppressive role of miR-29c in the development of hepatocellular carcinoma. The ectopic expression of miR-29c significantly suppresses liver carcinoma cell proliferation and induces apoptosis and cell cycle arrest (Figure 3). In contrast, the knockdown of miR-29c promotes liver carcinoma cell growth and inhibits cell apoptosis.

Although we (and other authors) have clearly demonstrated the suppressive role of miR-29c in liver carcinoma, the underlying mechanisms (key target molecules of miR-29c, in particular) remain largely unknown. This study has, for the first time, revealed that *WIP1* is a direct target of miR-29c, and it has also disclosed an inverse correlation between miR-29c expression and WIP1 levels in liver carcinoma cell lines and tissues. We have shown that the ectopic expression of miR-29c reduces the levels of WIP1; as a result, the phosphorylated p53 at Ser-15 is elevated in HepG2 cells (Figure 3H, the left panel). This may contribute to miR-29c-induced apoptosis and cell cycle arrest (Figure 3C and 3D) [39]. Conversely, miR-29c inhibitor increases the expression of WIP1, thus leading to a reduction in the phosphorylated p53 at Ser-15 (Figure 3H, the right panel). These findings are consistent with previous reports [32]. In addition to miR-29c, another well-defined tumor suppressor, miR-34a, is also transcriptionally regulated by the phosphorylated p53 at Ser-15 [40], suggesting an important role of p53 Ser-15 phosphorylation in transcriptional regulation of tumor suppressor miRNAs. Although this is the first description of miR-29c in the maintenance of p53 activity via targeting WIP1, other studies have indicated that miR-29 may also activate p53 by targeting p85 alpha and CDC42 [41]. As expected, the enforced expression of miR-29c induced the expression of p53 transcriptional targets p21 and p27 (Figure 3H, the left panel), the cyclin-dependent kinase inhibitors, due to the upregulation in p53 Ser15 phosphorylation [42]. This may contribute to the miR-29c-induced G1 arrest [43] (Figure 3D). Interestingly, the ectopic expression of miR-29c also caused an elevation in cleaved caspapse-3 with no effect on BAX (Figure 3H, the left panel), although a Bax-dependent caspase-3 activation has been shown to be essential for p53-induced apoptosis in neurons [44]. The upregulation of cleaved caspase-3, however, may play a crucial role in miR-29c-induced apoptosis (Figure 3C). Unlike p21 and p27, a miR-29c inhibitor remarkably attenuated BAX expression (Figure 3H, the right panel) due to the reduction in p53 Ser15 phosphorylation. The downregulated BAX may contribute to the miR-29c inhibitor-mediated reduction in apoptosis (Figure 3G) through the activation of caspases other than caspase-3, for instance, capase-7 [45]. To date, many other oncogene mRNAs have been identified as targets of miR-29c, such as *cyclin D2* (*CCND2*) [17], *matrix metalloproteinase-2* (*MMP2*) [17, 18, 21], *integrin β1* [18], mammalian *SIRT1* [22], *BCL2* [23], *MCL1* [23], *T-cell lymphoma invasion and metastasis 1* (*TIAM1*) [25], and *cyclin E* [27]. Interestingly, recent studies have found that miR-29c enhances the sensitivities of human nasopharyngeal carcinoma to both chemotherapy and radiotherapy [46]. miR-29 has also been linked to aging. Recently, a comprehensive study indicated an miR-29-induced cellular senescence in aging muscles through targeting multiple signaling pathways, including p85a, IGF-1 and B-myb [47].

In addition to p53, WIP1 can also dephosphorylate several other DNA damage-responsive proteins, including those of the ataxia-telangiectasia mutated gene (ATM), the ataxia-telangiectasia and Rad3-related protein (ATR), checkpoint kinase 1 (CHK1), checkpoint kinase 2 (CHK2), and p38 MAPK [48]. Due to its distinctive oncogenic properties mediated by inhibitory functions on the above mentioned tumor suppressor pathways [49], WIP1 has been linked to cancer. *WIP1* is frequently amplified and overexpressed in neuroblastoma, medulloblastoma, breast, pancreatic, ovarian, and gastric carcinomas [50–55]. WIP1 was overexpressed in 45.4% of our liver carcinoma tissue samples, whereas miR-29c was downregulated in 50.6% of the samples (Figure 4A and 4B). We have concluded that the expression level of miR-29c is inversely correlated with that of WIP1 in both normal liver and carcinoma tissues (Figure 4A and 4B). For the first time, we have established a causal relationship between the downregulation of miR-29c and the upregulation of WIP1 in liver carcinomas. We have also noted that the percentage of liver carcinoma tissues with WIP1 overexpression increases slightly with the progression of this disease (Figure 4D), implicating that the overexpressed WIP1 may contribute primarily to the development of liver carcinoma. Over half of human malignancies contain mutations in p53, including 29% HCC reported in Japanese patients (*n* = 169) [56]; however, a sizable fraction of HCC carries wild-type p53. Our results may imply an important role of the overexpressed WIP1 in liver carcinomas with wild-type p53 via inactivating p53 functions. These findings make WIP1 a potent therapeutic target against HCC. Although this is the first report of the existence of an inverse correlation between cellular levels of miR-29c and WIP1, the downregulation of miR-29c has been found in medulloblastoma [57], glioma [58] and gastric cancer [59]; however, the upregulation of WIP1 has also been reported in these carcinomas [60–62], implicating a possible inverse correlation between these two molecules. Further studies are needed to dissect the role of miR-29c and WIP1 in carcinogenesis and cancer.

**Figure 5:**
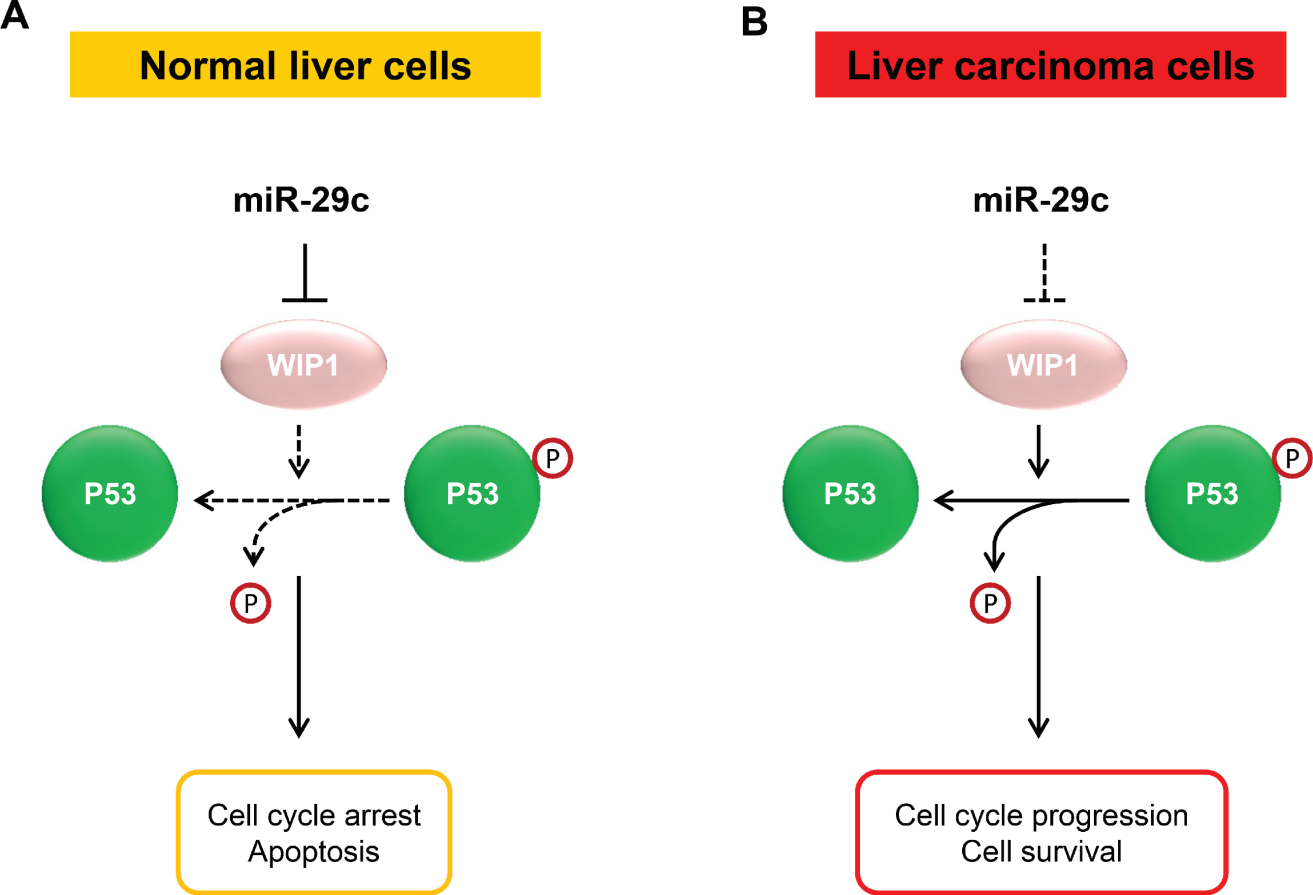
A model for the suppressive role of miR-29c in liver carcinoma **(A)** In normal liver cells, the normally expressed miR-29c may contribute to the maintenance of normal p53 activity via targeting *WIP1* phosphatase, thus maintaining a normal growth, proliferation, and apoptosis. **(B)** In liver carcinoma cells, miR-29c is downregulated due to promoter hypermethylation, leading to the overexpression of WIP1 that inactivates p53 by dephosphorylating p53 at Ser-15, which results in the promotion in cell proliferation and reduction in apoptosis that may contribute to the development of liver carcinoma.

In summary, low-dose IR-responsive miR-29c is downregulated in liver carcinoma cells and tissues. miR-29c functions as a tumor suppressor that plays a crucial role in the development of hepatocellular carcinoma via targeting *WIP1* (Figure 5A and 5B), and it may represent a target molecule for therapeutic intervention for this disease.

## MATERIALS AND METHODS

### Animal irradiation

Male and female 8-week-old C57BL/6 mice purchased from Charles River Laboratories (Quebec, Canada) were randomly assigned to different treatment groups. The control group was sham-treated (0/0), and the treatment groups were characterized as follows: low-dose group (0.3/0)—mice were exposed to only 0.3 gray (Gy) irradiation on day 0; high-dose group (0/3)—mice were exposed to only 3 Gy acute irradiation on day 4; priming group (0.3/3)—mice were exposed to both 0.3 Gy priming irradiation on day 0 and 3 Gy acute irradiation on day 4. Twenty mice per group (10 males and 10 females) were sacrificed at 6 hours, 96 hours, and 2 weeks after irradiation. Liver specimens were frozen immediately and stored at –80°C or fixed in 10% neutral buffered formalin and embedded in paraffin. Handling and care of animals were in accordance with the recommendations of the Canadian Council for Animal Care and Use. Procedures were approved by the University of Lethbridge Animal Welfare Committee. Animals were housed in a virus-free facility and given food and water *ad libitum*.

### MicroRNA profiling

Three replicates were used for each group described in the “Animal irradiation” section. Total RNA was isolated from the liver tissues of IR-exposed mice at 96 hours post IR using TRIzol reagent (Invitrogen, San Diego, CA) according to the manufacturer's instructions. MicroRNA profiling, clustering and data analysis were carried out by LC Sciences (Houston, USA).

### Cell culture

Human embryonic kidney 293 (HEK293) cells were grown in DMEM/High Glucose (Thermo Scientific, Waltham, MA) supplemented with 10% fetal bovine serum (FBS) and 1% penicillin/streptomycin (P/S). Human hepatocellular carcinoma cell lines HepG2 and C3A were cultured in ATCC-formulated EMEM (ATCC, Manassas, VA) supplemented with 10% FBS and 1% P/S. The normal mouse liver epithelial cell line NMuLi and the mouse hepatoma cell line Hepa 1–6 were grown in ATCC-formulated DMEM (ATCC) supplemented with 10% FBS and 1% P/S. All cells were cultured at 37°C in a humidified atmosphere of 5% CO_2_.

### MicroRNA real-time RT-PCR

Total RNA isolated from liver tissue of mice exposed to IR, normal human liver tissue (Amsbio, Cambridge, MA), NMuLi, Hepa 1–6, HepG2, and C3A cell lines was subjected to real-time RT-PCR using the miScript II RT Kit (QIAGEN, Chatsworth, CA), miR-29c miScript primer assays, and the miScript SYBR^®^ Green PCR Kit per the manufacturer's instructions. RNU6–2 was used as a loading control.

### The generation of luciferase reporter plasmid constructs

Luciferase miR-29c target reporters were generated as described previously [12]. Briefly, oligos corresponding to portions of the 3′UTRs of *WIP1* were synthesized, annealed, and cloned downstream of the luciferase gene in the pGL3-Basic vector between *Xba* I and *EcoR* I (a linker introduced by Mr. James Meservy) to generate Luc-WT-WIP1 and Luc-MT-WIP1 reporters. The sequence identity was confirmed by automatic sequencing. Oligo sequences were as follows: *WIP1* 3′UTR-WT1: 5′-/5Phos/CTA GAC TTG CTA TTT CAA TAA CAG ATG GTG CTG CTG-3′, *WIP1* 3′UTR-WT2: 5′-/5Phos/AAT TCA GCA GCA CCA TCT GTT ATT GAA ATA GCA AGT-3′ *WIP1* 3′UTR-MTa: 5′-/5Phos/CTA GAC TTG CTA AAA AAA TAA CAG AAA AAA AAG CTG-3′, *WIP1* 3′UTR-MTb: 5′-/5Phos/AAT TCA GCT TTT TTT TCT GTT ATT TTT TTA GCA AGT-3′.

### The transient transfection and luciferase assay

HEK293 cells grown to 90% confluence in 6-well plates in antibiotic-free DMEM/High Glucose (Thermo Scientific) containing 10% FBS were transiently cotransfected with either 0.5 μg of Luc-WT-WIP1 or Luc-MT-WIP1 reporter, 5-ng pRL-TK plasmid, and the indicated concentration of hsa-miR-29c mimic (QIAGEN) using Lipofectamine 3000 (Invitrogen) according to the manufacturer's instructions. Twenty-four hours after transfection, the cells were harvested, the relative luciferase activity was measured by the Dual-Luciferase Reporter Assay System (Promega, Madison, WI) using a luminometer (FLUOstar Omega, BMG LABTECH, Germany) and the wild-type *Firefly* luciferase data were normalized to mutant *Firefly* luciferase.

### The transient transfection and MTT assay

HepG2 cells grown to 90% confluence were transiently transfected with either 100 nM miR-29c mimic, 100 nM miR-29c inhibitor, or 100 nM AllStars negative control (QIAGEN) using Lipofectamine 2000 (Invitrogen) according to the manufacturer's instructions. Twenty-four hours after transfection, 3.0 × 10^3^ HepG2 cells were plated in 96-well plates. The 3-(4,5-Dimethylthiazol-2-yl)-2,5-diphenyl tetrazolium bromide (MTT) assays were carried out using a Cell Proliferation Kit I (Roche Diagnostics GmbH, Mannheim, Germany) according to the manufacturer's instructions. The spectrophotometric absorbance of samples was measured at 595 nm using a microtiter plate reader (FLUOstar Omega).

### Cell cycle and apoptosis analyses

HepG2 cells grown to 90% confluence were transiently transfected with either 100 nM miR-29c mimic, a 100 nM inhibitor, or 100 nM AllStars negative control. At 96 hours post-transfection, the cells were harvested for cell cycle and apoptosis analyses that were performed with a BD FACSCanto™ II Flow Cytometer (BD Biosciences, Franklin Lakes, NJ) using a propidium iodide staining solution and a BD Pharmingen™ V-FITC Annexin Apoptosis Detection Kit II (BD Biosciences) according to the manufacturer's instructions.

### Bioinformatics

Potential hsa-miR-29c targets were predicted by both TargetScan and RNAhybrid software applications. The correlation between miR-29c and WIP1 was predicted using miRGator (http://mirgator.kobic.re.kr/index.html).

### Western blot analysis

NMuLi, Hepa 1–6, C3A, and HepG2 cells or HepG2 cells transfected with either 100 nM miR-29c mimic, a 100 nM inhibitor, or 100 nM AllStars negative control were washed twice with ice-cold PBS and lysed in radioimmunoprecipitation assay buffer (RIPA). Protein isolated from human normal liver tissue (HNLT) purchased from AMSBio (AMS Biotechnology, Cambridge, MA) served as a normal control for human cell lines. 30–60 μg of protein per sample was electrophoresed on either 8% or 10% SDS-PAGE and electrophoretically transferred to a polyvinylidene difluoride (PVDF) membrane (Amersham Hybond^®^ P, GE Healthcare, Buckinghamshire, UK) at 4°C for 1.5 hours. Blots were incubated for 1 hour with 5% nonfat dry milk to block nonspecific binding sites, and then they were incubated overnight at 4°C with polyclonal/monoclonal antibodies against BAX, BCL2, MCL1, SIRT1, p27, p–p53 (Santa Cruz Biotechnology, Dallas, TX) or cleaved caspase 3, p21, p53 (Cell Signaling Technology, Danvers, MA), AGO2, or WIP1 (Abcam, Cambridge, UK). The immunoreactivity was detected using a peroxidase-conjugated antibody and visualized by an ECL Plus Western Blotting Detection System (GE Healthcare). The blots were stripped before reprobing with an antibody to actin or GAPDH (Santa Cruz Biotechnology).

### Fluorescence *in situ* hybridization

Hsa-miR-29c expression in liver carcinoma specimens (LVC481 and LVC2281 tissue arrays; Pantomics, Richmond, CA) was determined by FISH, as detailed elsewhere [63]. After deparaffinization, the sections were prehybridized for 20 minutes at 55°C followed by 1 hour hybridization at the same temperature with a 1:1000 dilution of a miRCURY LNA™ hsa-miR-29c detection probe (Exiqon, Vedbaek, Denmark). After washing, the sections were blocked for 1 hour with a blocking solution and incubated with a 1:1000 dilution of anti-Digoxigenin-Fluorescein, Fab fragments (Roche, Basel, Switzerland) at 4°C overnight. Then research scientists and a pathologist independently analyzed the stained tissue sections.

Staining intensity was the criterion used for quantitating immunofluorescence staining. A range of 0 to 3 was used for classifying the intensity: 0 = absence of staining; 1 = weak staining; 2 = moderate staining; and 3 = intense staining.

### Immunohistochemical analysis

The expression of WIP1 in the liver carcinoma specimens (LVC481 and LVC2281 arrays; Pantomics) was determined by immunohistochemical staining using a mouse monoclonal antibody to WIP1 (Santa Cruz Biotechnology, Dallas, TX) according to the Biocare Medical instructions for immunohistochemistry. The stained tissue sections were analyzed independently by a pathologist and research scientists in a blind manner.

The criteria used for quantitating immunohistochemical staining included the staining intensity and percentage of cells stained. A range of 0 to 3 was used to classify the intensity of staining: 0 = absence of staining; 1 = weak staining; 2 = moderate staining; and 3 = intense staining. The numbers of cells stained were recorded according to the following classification: a, < 25% of cells stained; b, 25%–50% of cells stained; c, 51%–75% of cells stained; and d, > 75% of cells stained.

### Statistical analysis

The Student's *t*-test was used to determine the statistical significance of differences between groups in hsa-miR-29c and WIP1 expression, cell growth, cell cycle, apoptosis, luciferase activity, and the correlation of WIP1 with HCC clinical grades. The Pearson correlation was used to determine the statistical significance in has-miR-29c and WIP1 expression between normal and tumor tissues. *p* < 0.05 was considered significant.

## SUPPLEMENTARY FIGURE


