# Supplementary material for: A suppressive role of ionizing radiation-responsive miR-29c in the development of liver carcinoma via targeting WIP1
Source: Oncotarget. 2015 Apr 4;6(12):9937–50. doi: 10.18632/oncotarget.3157 (PMC4496408; doi:10.18632/oncotarget.3157)
Supplement: Supplementary file 1 [file oncotarget-06-9937-s001.pdf]

SUPPLEMENTARY FIGURE

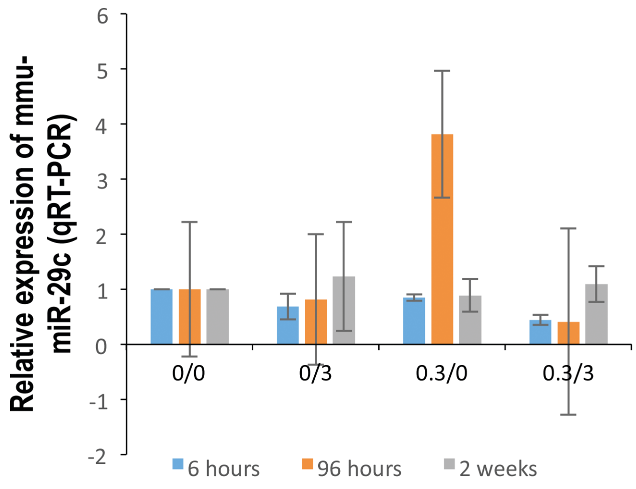

Supplementary Figure S1: miR-29c differentially expressed in IR-exposed mouse liver. 8-week-old female mice were randomly assigned to different groups, sham-treated group (0/0), low-dose group (0.3/0), high-dose group (0/3), and priming group (0.3/3) and exposed to IR as described in “Materials and Methods”. The IR-exposed mice were sacrificed at 6 hours, 96 hours, and 2 weeks after irradiation. Total RNA isolated from mouse liver tissues was subjected to qRT-PCR using mmu-miR-29c primer set.
